# A Novel Re-keying Function Protocol (NRFP) For Wireless Sensor Network Security

**DOI:** 10.3390/s8127866

**Published:** 2008-12-04

**Authors:** Maan Younis Abdullah, Gui Wei Hua, Naif Alsharabi

**Affiliations:** 1 School of Information Science and Engineering; Central South University / Hunan, ChangSha, 410083, P. R. China; E-Mail: gwh@csu.edu.cn; 2 Hunan University / Hunan, ChangSha, 410082, P. R. China; E-Mail: sharabi28@hotmail.com

**Keywords:** Session key, Re-keying function, Session key derivation

## Abstract

This paper describes a novel re-keying function protocol (NRFP) for wireless sensor network security. A re-keying process management system for sensor networks is designed to support in-network processing. The design of the protocol is motivated by decentralization key management for wireless sensor networks (WSNs), covering key deployment, key refreshment, and key establishment. NRFP supports the establishment of novel administrative functions for sensor nodes that derive/re-derive a session key for each communication session. The protocol proposes direct connection, in-direct connection and hybrid connection. NRFP also includes an efficient protocol for local broadcast authentication based on the use of one-way key chains. A salient feature of the authentication protocol is that it supports source authentication without precluding innetwork processing. Security and performance analysis shows that it is very efficient in computation, communication and storage and, that NRFP is also effective in defending against many sophisticated attacks.

## 1. Introduction

Sensor networks can consist of hundreds or even thousands of sensor nodes, low power devices equipped with one or more sensors. Providing security is particularly challenging in sensor networks due to the resource limitations of sensor nodes.

Many research issues arise from such challenges in NRFP. For example, allowing multiple key functions in the sensor network adds to the robustness of the sensor network but it also makes the key management protocol different from that used in WSNs. Thus, key management protocols for sensor networks are based upon symmetric key algorithms.

Many sensors systems are deployed in unattended and often adversarial environments. Hence, security mechanisms that provide confidentiality and authentication are critical for the operation of many sensor applications. A sensor node typically contains signal processing circuits, microcontrollers, and a wireless transmitter/receiver. These components, if implemented without any security, could easily become a point of attack. Security must therefore pervade every aspect of the design of a wireless sensor network application that will require a high level of security [1]. By feeding information about the physical world into the existing information infrastructure, these networks are expected to lead to a future where computing is closely coupled with the physical world and is even used to affect the physical world via actuators. Potential applications include monitoring remote or inhospitable locations, target tracking in battlefields, disaster relief networks, early fire detection, and environmental monitoring. Despite sensors being used in many important applications, recent research has not focused adequately on the issue of security and protection, emphasizing instead on energy efficiency [2], network protocols [3] and distributed databases.

Sensor networks have distinctive features, the most important of which are constrained energy and computational resources. An important design consideration for security protocols based on symmetric keys is the degree of session key between the nodes in the system. At one extreme, there are network-wide keys that are used for encrypting data and for authentication. This key sharing approach has the lowest storage costs and is quite energy-efficient. However, it has the obvious security disadvantage that the compromise of a single node reveals the global key.

Zhu *et al.*'s[4] Localized Encryption and Authentication Protocol (LEAP) is a complete key management framework for static WSNs. It includes mechanisms for securing node-to-base station traffic, base station-to-nodes traffic, local broadcasts and node-to node (pair-wise) communications.

In this study, NRFP is described as a sensor network security re-keying function protocol that is designed to support decentralized key management architecture for WSNs, while providing security properties similar to those provided by session key schemes. The premise of NRFP is that no single keying mechanism is appropriate for all of the secure communications that are needed in sensor networks. As such, NRFP supports the establishment of three types of keys for each sensor node: a Master key (M_K_) shared by all the nodes in the network; a Local key (L_K_) shared with the base station; and a Session key (S_K_) shared with another sensor node. It is proposed that the local administrative functions (LAFs) acting as master function, re-keying function, and derivation function be imprinted with sensor node to achieve a high-level security of node-to-node communication. The derivation function is used to generate new key values based on a request message which comes from the base station (BS) or cluster head (CH). In other words, the keying mechanisms provided by NRFP enable in-network processing, while restricting the security impact of a node compromise to the immediate network neighborhood of the compromised node. NRFP architecture uses only symmetric-key cryptography, and is based on a clear set of assumptions and guidelines.

The rest of this paper is organized as follows. A review of previous work is covered in Section 2. Section 3 discusses design goals and assumptions. The NRFP protocol is presented in detail in Sections 4, 5 and 6. Section 7 analyzes the performance and security of the NRFP protocol. A prototype implementation of NRPT is reported in Section 8.

## 2. Related Work

The following discusses some of the most important work in the literature in terms of key management protocol. Basagni *et al.*'s pebblenets [5] are tailored to sensor nodes that have severe computational and storage limitations but are tamper-resistant.

Perrig *et al.*'s security protocol for sensor networks (SPINS) [6] is a centralized architecture that assumes a tree-like network topology. At the root of the tree is a base station. Sensor nodes form the rest of the tree. SPINS has two building blocks: secure network encryption protocol (SNEP) and the micro version of the timed efficient, streaming, loss tolerant authentication protocol (μTESLA).

Eschenauer *et al.* [7] pioneered random pre-distribution schemes. The basic scheme is best studied from two perspectives, random graphs and combinatorics. Chan *et al.* [8] and Di Pietro *et al.* [9] propose several improvements to Eschenauer *et al.*'s basic scheme.

LEAP, LEAP+, and LEAP++ include support for establishing four types of keys per sensor node– individual keys shared with the base station, pairwise keys shared with individual neighboring nodes, cluster keys shared with a set of neighbors, and a global key shared by all the nodes in the network. These keys can be used to increase the security of many protocols. LEAP+ can prevent or increase the difficulty of launching many security attacks on sensor networks. The key establishment and key updating procedures used by LEAP+ are efficient and the storage requirements per node are small. LEAP++ achieves better node compromise resilience with one-time use master keys within shorter time intervals and provides enough resistance against DoS attacks and node fabrication attacks. It also has a distinguishing feature such that node replication and wormhole attacks can be easily detected in many cases, if not all. But all LEAP models are static and cannot solve broadcast all keys.

Zhang *et al.* [12] developed a fast verification approach with the help of roadside units (RSUs). When a vehicle enters into an RSU's transmission range, the RSU assigns a unique shared symmetric key and a pseudo identity to this vehicle. The vehicle generates a symmetric MAC code using this symmetric key, and then broadcasts each message by signing the message with the symmetric MAC code instead of a PKI-based private key.

Key management is one of the oldest areas in WSN security; many studies have been conducted, and key management schemes have been improved. At the beginning, we have Basagni *et al.*'s pebblenets that requires tamper resistance, which is actually something to be avoided in WSNs. Then we have Perrig *et al.*'s centralized architecture, SPINS. Zhu *et al.*'s LEAP provides explicit support for all predominant forms of communication typical of WSNs, but it only works for static networks. The disadvantage is that it depends upon RSUs. In reality, RSUs are not located everywhere and the roads are not fully covered by the communication range of RSUs. This scheme cannot function in areas without RSU.

## 3. Security Assumptions and Goals Design

NRFP presents a new methodology in keying information for wireless sensor nodes to insure the secure communication between nodes on the networks topology. NRFP is designed to improve the cluster formation security of key management [4-8], and the proposal is designed to support secure communications in sensor networks; therefore, it provides the basic security services such as confidentiality and authentication. In addition, NRFP is to meet several security and performance requirements that are considerably more challenging to sensor networks.

A dynamic sensor network is used. The base station, acting as a controller (or a key server), is assumed to be supplied with long-lasting power. The sensor nodes are similar in their computational and communication capabilities and in power resources to the current generation sensor nodes. The sensor nodes can be deployed via aerial scattering or by physical installation. Regarding security and goal design, a number of logical assumptions need to be made: the immediate neighboring nodes of any sensor node will not be known in advance. Because wireless communication is not secure, an adversary can eavesdrop on all traffic, inject packets, or replay older messages, if a node is compromised, all the information it holds will be known to the attacker. However, the base station will not be compromised; the physical layer of a wireless sensor network could use techniques such as spread spectrum [9] to prevent physical jamming attack if necessary. Techniques such as ALOHA and Slotted ALOHA [16] may be used to relieve attacks on the underlying media access control protocol.

## 4. Novel Re-keying Function Protocol (NRFP) and Authentication

### 4.1. Re-keying Function Protocol (NRFP)

**Figure 1.**
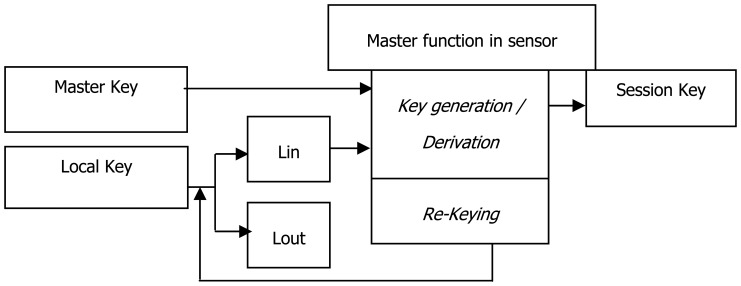
Novel Re-keying Function Protocol (NRFP).

The sensor nodes (Figure 1) should have the following keys: M_K_, which is shared by all the nodes in the network; L_K_, which is shared with the BS; and S_K_, which is shared with another sensor node. Each of these keys is considered in turn with the reasons for including it in the prototype.

**Master key (M_K_)**: This is a globally shared key that is used by the base station for encrypting messages that are broadcast to the whole group. Each sensor node is imprinted with master key and LAFs when it is manufactured.

**Local key (L_K_)**: Every node has a unique key that is injected with initial local key (L_K_), is shared with the base station. This key is the basic parameter for the re-keying function of the proposal and is used for secure communication between the node and the base station.

**Session key (S_K_)**: Every node shares an S_K_ with each of its immediate neighbors. In NRFP, S_K_s are used for securing communications that require privacy or source authentication.

**LAFs**: The local administrative functions include ‘master function’, ‘re-keying function’, and ‘derivation function’ and can be imprinted with sensor node to achieve a high-level security of node-to-node communication. The LAFs are responsible for key generation of the cluster session keys depending on which initial master key and local control key were imprinted at the time of manufacturing, whereas the HMAC is adopt of LAFs work. Master function, the derivation function is used to generate new key values based on requesting message coming from BS or CH. The re-keying process is necessary for two reasons:

1) It is simple for k to compute f(k), but computationally infeasible for f(k) to compute k.
2) k0, k1, k2,…kn, are computationally infeasible to compute f(k), as long as it is computationally infeasible to compute k.

Prior to node deployments each node is injected with initial L_K_, which is the basic parameter for the re-keying function of our proposal. The re-keying function is responsible for assigning a new value to L_K_. The cluster head periodically refreshes to respond to the changes of the L_K_ key, which notifies all of its members in a secret way of the new change. Functions and keys implement through the fundamental principles of the key management as following:

1) Key deployment: every node is imprinted with unique ID, M_K_, L_K_ and master functions that can generate and regenerate the unique sharing key with other nodes driving from M_K_ and L_K_. Those keys that were imprinted never exchange during a communication session between nodes; the only key exchanged to establish communication between two nodes is the S_K_.
2) Key Establishment: all nodes on the network use the same mechanism to communicate securely with each other. After deployment and the completion of the cluster head performance, the cluster head generates the S_K_ and sends the control message to its members to encourage them to generate the S_K_. Intra-cluster node-to-node communications are supported for this round: when two nodes want to communicate with each other they use the same S_K_ to establish secure communication and initiate the exchange of data. S_K_ should be the same because all nodes are using the same derivation function.
3) Node addition: when a new node joins the network, it first must join to any cluster on the network. If it receives a cluster head beacon, the key refreshment runs inter-cluster and generates its own S_K_. If a node does not receive any CH beacons, it becomes its own cluster and acts as a CH of this cluster, then runs the LAFs to generate its own keys.
4) Node eviction: node eviction means that any node in the cluster leaves its region for any reason (Power consumption, node emigration, node capture, etc.). In this case, we propose two cases of node eviction:
  Case 1: member node eviction occurs when the cluster head does not receive the hello message from a certain node, CH sends a hello message to that node and waits for a reply. If it does not receive a reply within a certain time, the cluster head sends a message to all of its members to inform them to delete the node with a certain ID from the list of neighbors.
  Case 2: in CH eviction when a cluster head leaves the cluster, two processes must be completed. First, the cluster head sends messages to all of its members to inform them that it is going to leave. From each cluster member, the node members then elect the cluster head which has a highest number of a list of neighbors or the node that has the highest power. Second, if the cluster head left surreptitiously, the entire cluster member will not receive the CH beacon for a period, and then the cluster members rebuild the cluster according to cluster base process and elect a new cluster head.

### 4.2. Authentication

For a message authentication code (MAC) function a MAC algorithm can be generated using multiple different techniques, as long as the sender and receiver have shared secret keys. A MAC algorithm can create out of a common symmetric cipher such as DES2 or AES3. A sender wanting to send a secure message can send M encrypted, e(M), with a symmetric cipher and then resend M‖K (M concatenated with K) encrypted, e(M‖K). The receiver first decrypts M, d(e(M)), to generate M′. M′‖K, e(M′‖K) are then encrypted and compared with the e(M‖K) originally sent. If the two match, then this confirms that the data was not corrupted.

HMAC [13] is merely a specific type of MAC function. It works by using an underlying hash function over a message and a key. Any hashing function could be used with HMAC, although more secure hashing functions are preferable. Moreover, HMAC is computationally very fast and compact. HMAC accomplishes both of these properties because of its reliance on a given hash function which is fast and returns compact outputs.

## 5. NRFP models

Two systems models of re-keying process are suggested to improve the secure communication: base station model and cluster model. These two models rely on a process of re-keying session keys. They use a passive cluster head election scheme, the structure of which has two parts: these cluster head (CH) and cluster nodes. First, every sensor broadcasts its ID on a specific time shift before being embedded into the targeted area; then it listens to its neighbors, adds their IDs in its routing table, and calculates the number of messages it receives to find the number of neighbors (NBR) it can reach. These connected neighbors build their own group or cluster.

To determine the cluster head, sensors broadcast their IDs and NBRs. Every sensor keeps a list of all its neighbors' NBRs. A sensor becomes a cluster head if it has the highest NBR. We chose this approach because cluster heads receive more messages than other nodes. The cluster head with its connected neighbors form the cluster or the group. We call the cluster head's neighbors its “children” because the cluster head and its children have parent-child relationship in the tree-based network.

### 5.1. Base Station Model

In this model, the re-keying process is controlled by the BS, by sending a message to all cluster heads in the network to encourage them to reconstruct a cluster and derive a new S_K_. This S_K_ is common for all nodes in the network. This operation is carried out in a regular and systematic time, so all nodes derive the S_K_ on the same time and continue establishing communication with each other. This centralized model is needed for re-keying process, scalability to extend the network size, and different applications that do not require the preparation of a large number of keys.

### 5.2. Cluster Head Model

This model is similar to the previous model in the process of derivation, but differs in that each of the clusters is separate on the timing of re-keying the derivation process. Each cluster has its own session key different from others. At the end of the restructuring cluster and when the session key has been performed, the cluster head derived shared key is sent to the base station where it is used in future communication between clusters. What distinguishes this model is that every independent cluster alone is re-building the key according to cluster characteristics; in the case of session key obtained, it does not affect the rest of the other clusters.

## 6. NRFP Connection

**Figure 2.**
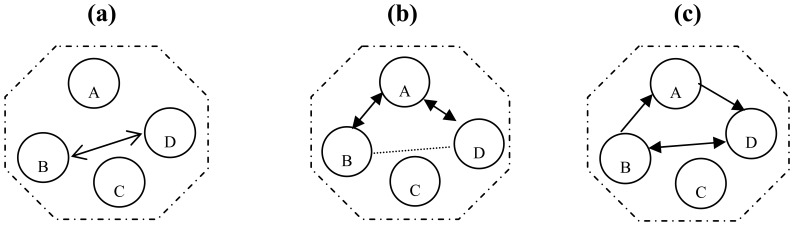
Clustering Connection Protocol (a) Cluster S Direct connection; (b) Indirect connection; (c) Hybrid connection

This section describes two scenarios of NRFP keying organizing protocol: NRFP base station re-keying algorithm and NRFP cluster head re-keying algorithm. These two scenarios are used to support two kinds of network connection protocols called intra- and inter-clusters. Suppose that node A is a cluster head of cluster S; B and D are nodes member of S cluster; B needs to connect to D. We suggest three protocols for these processes are direct connection protocol, indirect connection protocol and hybrid connection protocol (see Figure 2).

### Intra Cluster keying Protocols

1) Direct Connection Protocol is when two nodes communicate with each other secretly using a session key. Supposing that A is the head cluster in cluster S direct connection (see Figure 2.a), and a node B (with session key K_B_ with A) wants to initiate a session key with D (which shares key K_D_ with A), B and D share a common group K_A_. Concerning the notation, NA represents a nonce emitted by A, NB represents a nonce emitted by B and so on. MAC keys derived from key K are respectively denoted by K′ and K″ and then the derived session for a direct connection protocol is simplified as:
  B➔ D: NB,D,MAC_K″B_(NB/D)
  B➔ B:MAC_K′D,B_(KA/B/D),E_K′B_(E_K′D_(K_A_)|K_BD_),MAC_K″B_(NB|D|E_K′B_ (K_A_)|K_BD_)
  D ➔ B: D, E_K′B_(K_AB_),MAC_K″ B_(ND|B|E_K′B_(K_DB_))
  B ➔ D: A: Ack,MAC_K″BD_(Ack)
  Therefore, node B and D share a session key K_BD_ = K_DB._.
2) Indirect Connection Protocol is when two nodes communicate with each other secretly using shared key session, which are created by cluster head node. If node A was head for cluster S (see Figure 2b), the deriving session for an indirect connection between B and D are described as the following:
  B➔ A: NB,D,MAC_k″B_ (NB/D)
  A➔ B: E_K′B_(E_K′D_(NA)/K_BD_),MAC_K″B_(NB/D/E_K′D_(NA)/K_BD_)
  B➔ D: B,E_K′D_(NA)
  D➔ A: D,ND,B,MAC_K″D_(NA/D/ND/B)
  A➔ D: E_K′D_(K_BD_),MAC_K″D_(ND/B/E_K′D_(K_BD_))
  D➔ B: Ack, MAC_K″BD_(Ack)
3) Hybrid Connection Protocol uses almost the same technique of indirect protocol by using a head cluster to authenticate the communication between two nodes and a direct connection protocol for establish a S_K_ between those nodes, a simple description of this protocol shown on (see Figure 2.c) described as:
  B➔ A: N_B_,D,MACK″_B_ (N_B_/D)
  A➔ D: E_K′B_(E_K′D_(N_A_)/K_BD_),MAC_k″B_(N_B_/D/E_k′D_(N_A_)/K_BD_)
  D‐>B: D, E_k′B_(K_AB_),MAC_k″B_(N_D_|B|E_k′B_(K_DB_))
  B‐>D: A: Ack,MAC_K″BD_(Ack)

### Inter-Cluster Tree-based connection protocol

**Figure 3.**
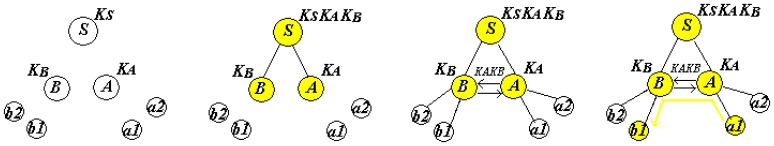
Direct Connection Protocol.

**Figure 4.**
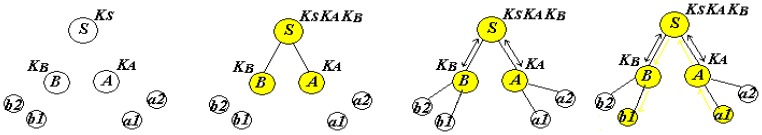
Indirect Connection Protocol.

**Figure 5.**
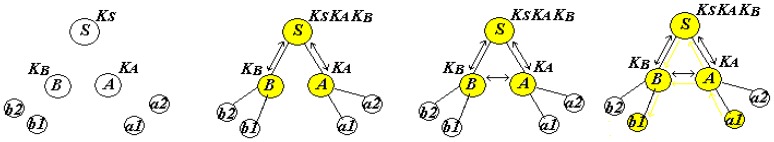
Hybrid mutual trust.

The security requirements of contributory key agreement between two groups (clusters) on the networks; in case of network depends on clusters independent self-organizing. The network is divided into levels that the upper level has local session key of its down levels, those levels classified as parents and children. The NRFP keying algorithm in this case will assume that the cluster is as a whole network proposed in the previous sections. Shared key of two or more clusters is generated by the parent by merging local session keys generated by children clusters. Three protocols proposed to perform the secure session between different clusters according to the network topology are direct, indirect, and hybrid connection as shown in Figures 3, 4 and 5:

#### 1) Direct Connection Protocol

Let *A* and *B* denote two subgroups (clusters) (see figure 4), *K_A_* is the common share key for all member in cluster A, and K_B_ is the common share key for all member in cluster B. Let *f* (K) (referred to as the blinded key of key K) denote the modular exponentiation operation, that is:

(1) f(k)=gK mod p

Here *g* is the exponential base and *p* is the modular base. Suppose that *A_1_* in cluster *A* needs to establish a connection session with *B1* in cluster *B*. *A* and *B* need to exchange the following keying messages if *A* and *B* on the same range: A sends the key f(K_A_) to *B*, and *B* sends the key f(K_B_) to all members of subgroup A. Now each member in *A* or *B* calculates the new group key K_AB_ as follows:

(2) $${\text{K}}_{\text{AB}}=({\text{f}(\text{K}}_{\text{B}})){\text{K}}_{\text{A}}\mathrm{mod}\text{p}{=(\text{f}(\text{k}}_{\text{A}})){\text{K}}_{\text{B}}\mathrm{mod}\;\text{p}$$

Refer to the assumption that each node has to be imprinted with a master key, L_K_ key and the re-keying function which gives the nodes on the network the abilities to key, re-key and derive keys.

A_1_➔ A: N_A1_,B_1_,MAC_K″A1_(N_A1_/K_A1_)
A➔ B:N_A_,N_A1_,B,B_1_,MAC_K″A_(N_A1_,N_A_, A1,A)
B ➔ B: N_A_, N_B_, A, B,MAC_K″B_(N_A_|N_B_|A|B)
Self-generation : E_K′B_(K_AB_),MAC_K″B_(N_B_|A|E_K′B_(K_AB_))
B➔ A: Ack,MAC_K″AB_(Ack)

#### 2) Indirect Connection Protocol

Figure 4 illustrates the process of establishing a secure session key between *A* and *B* using the higher level node S [5].Tree-based indirect connections indicate that two clusters in the same level can use the third party to certify and establish the session key, that the third party may be the server or the higher level of these two clusters.

A_1_➔ A : N_A1_,B/B1
A ➔ S_A_ : N_A_, B,MAC_K″A_ (N_A_|B)
S_A_➔ B : B, N_SA_ E_K′SS_(N_SA_),MAC_K″A_(N_A_|B|E_K′SS_(N_SA_))
B ➔ S_B_ : B, N_B_, A, E_K′SS_(N_SA_),MAC_K″B_(B|N_B_|A|E_K′SS_(N_SA_))
S _B_➔ S_A_: N_SB_, A, B, E_K′AB_(K_AB_), MAC_K″AB_(N_SA_|N_SB_ |A|B|E_K′AB_(K_AB_))
S_A_➔ S_B_: E_K′AB_(K_AB_),MAC_K″AB_(N_SB_ |A|B|E_K″AB_ (K_AB_))
S_A_➔ A : E_K″A_(K_AB_),MAC_K″A_(N_A_|B|E_K″A_(K_AB_))
S_B_➔ B : E_K′B_(K_AB_),MAC_K″B_(N_B_|A|E_K′B_(K_AB_))B ➔ A : Ack,MAC_KAB_ (Ack)

Where K_S_ is intuitively the shared key between S_A_ and S_B_, and K_AB_ = K_BA_ is the final established session key.

#### 3) Hybrid mutual trust

In Figure 5, for the hybrid mutual trust protocol two phases, direct connection and indirect connection, are performed. A simple description of this protocol is described as:

A_1_➔ A: N_A1_,B_1_,MAC_K″A1_(N_A1_/K_A1_)
A➔ B: N_A_,N_A1_,B,B_1_,MAC_K″a_(N_A1_,N_A_, A_1_,A)
B ➔S: N_A_, N_B_, A, B,MAC_K″B_(N_A_|N_B_|A|B)
S ➔ B: E_K′SB_(K_AB_),MAC_K′SB_(N_B_|A|E_Kk′SB_(K_AB_))
B➔A: Ack,MAC_K″AB_(Ack)

The above proposed protocol shows that cluster *A* and cluster *B* can generate a sharing secure session key K_AB_=K_BA_, then cluster A send K_AB_ to all A's nodes members and Cluster *B* send K_BA_ to all B's nodes member. Therefore, each node in cluster A can establish secure connection with other nodes in cluster *B* and start exchanging the secure data according to the secure key session generated between A's and B's nodes.

## 7. Security and Performance Analysis

In this section we first discuss the security of the protocol. Then, we analyze the computation, communication costs and storage requirement

### 7.1. Security analysis

Our proposed key management protocol NRFP satisfies the following properties:

Property 1: Only the authorized sensors can communicate in the network. The communication among the sensors is ensured by the M_K_, L_K_, and S_E_. Unauthorized sensors (outside attackers) cannot participate in the communication without proper assigned key materials.
Property 2: The session key distribution process is secure. The distribution of session key is based on the personal key share distribution scheme [4]. A revoked sensor cannot recover the session key because of the key to self-generation and thus does not need to deploy Log. Because of the broadcast, an outside attacker cannot masquerade as a base station disseminating a session key and start a revocation attack either.

If the session *k'* key is compromised for any reason and an adversary attacker can capture these keys, it is infeasible to deduce *k* from it because one of the parameter of this key is not found and already assigned to a new value. This is one advantage of NRFP re-keying proposal. An attacker needs to know three parameters to break the link layer security M_K_, L_K_, S_K_ and re-keying function, that is responsible for keying and re-keying keys session for inter and intra cluster communication method. This makes NRFP re-keying algorithm very complicated for an attacker to attack the link layer communication on the networks even if he has some how discovered a key session. Secure hash functions, such as SHA-1 [14] or SHA-2 [15], are good candidates for the key derivation function.

### 7.2. Performance analysis

Because base stations are usually regarded as resource-rich nodes, we focus on the performance of sensor nodes:

1) Computation cost: there are three different types of keys in our proposed protocol, namely the M_K_, L_K_ and S_K_. To calculate these keys, polynomial evaluation is required. The computation of polynomial evaluation is efficient. The calculation of the encryption key and the MAC key is based on a pseudo-random function. Thus, the key distribution scheme is efficient in computation.
2) Communication cost: the communication cost includes the setup of the M_K_, L_K_, and S_K_. Because the keys self-generate and do not require deployment of Log, the base station just sends a re-keying message. The session key is *not distributed* to the network and does not need a *broadcasting message*. The maximum size of the broadcast message need only be large enough to send a re-keying message, the propagation delay is very small.
3) Storage requirement: let d represent the number of neighboring nodes around a sensor. Each sensor node requires d storage units for the S_K_ keys, and three storage units for the session key, because of self- generation. The S_K_s for all sensor nodes do not need to exchange the key with other sensor nodes which were generated in with the same S_K_ at the same time, so d=0. Thus, the total storage units of keys required for each sensor is: unitsM_K_+unitsL_K_+unitsS_K_

## 8. Simulations and Discussion

The role-based hierarchical self organization protocol was simulated using C++. The simulator can also be used to view the topology generated by the initial self organization algorithm. A comparison was assumed to have the same number of clusters or sensing zones, no packet collisions occurred. It also assumed that there were no packet errors during transmission and reception.

In other words, we assumed a perfect wireless channel. Simulation runs with the following simulation parameters:

Number of nodes = 50 to 1,000;
Maximum X, Y boundary coordinates of a region of WSN deployment = 100 × 100 to 2,200 × 2,000 meters;
Maximum wireless radio range and sensing range = 90 meters;
Application specified sensing accuracy (d) = 8 meters.
Assume that the malicious nodes have the same energy as other nodes.

An initial performance evaluation of the network comparison with others previous works uses the OmNet++ simulator. The simulation was run in different scenarios, each scenario has different parameter values, malicious nodes inject with right key session in the beginning to be the same with the other nodes on the cluster. The proposed system must recognize these nodes and refuse them connection for next round. Nodes are deployed according to a uniform distribution function over an area from 100 × 100 meters to 2,200 × 2,000 meters. The node closest to the center of the deployment area is selected as sink, which is not resource limited, secure and safe from any advisory attackers.

For all the topologies, we set the radio range and the sensing range to 64 meters. The minimum and maximum sensing zone (or cluster) membership size was set to 5 and 12, respectively. Finally, the application specified sensing accuracy or the sensing cell dimension (d) was set to the values 8, 12, and 16 for the above simulation scenarios.

Assume an attacker has two goals: the primary goal is to disrupt the network by preventing messages from arriving at the sink node, and the secondary goal is to increase the energy wastage of the sensors. To simulate attacks, the malicious nodes are activated 10 seconds after the sensor network starts operating. They are activated by giving the session key during the phase composition of clusters, so that they may have ability to establish a connection session with other nodes within the cluster. These malicious nodes continue to work within the clusters until the network goes down or maintain by the network administrator.

**Figure 7.**
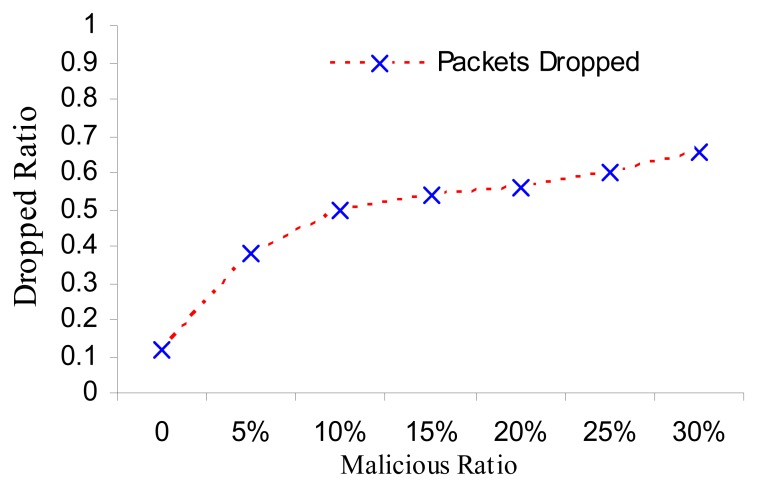
Selective forwarding attack run with seven scenarios implemented according to the number of malicious nodes on the network, the energy consumption of each sensor node [17] is as follows: Ea=100 pJ/bit/m2, Ee = 50 nJ/bit and Ec = 5 nJ/bit were consumed for transmitting, receiving and listening respectively each sensor needs to send a packet of length R = 400 bits to the cluster head on random time. Cluster head period T is set as 2,000s, and the execution time of task is set as = 0.005 s. The data packet size is 2 KB and the sensing range to 64 meters.

**Figure 8.**
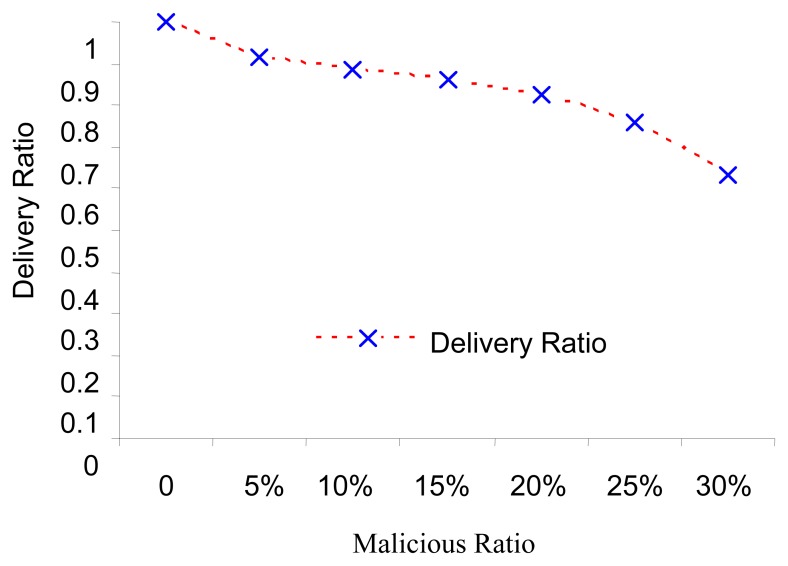
NRFP : Black Hole and Selective attacks run with seven scenarios implemented according to the number of malicious nodes on the network. The energy consumption of each sensor node [17] is as follows: Ea=100 pJ/bit/m2, Ee = 50 nJ/bit and Ec = 5 nJ/bit where consumed for transmitting, receiving and listening respectively each sensor needs to send a packet of length R = 400 bits to the cluster head on random time. Cluster head period T is set as 2,000 s, and the execution time of task is set as = 0.005 s. The data packet size is 2 KB and the sensing range to 64 meters.

**Figure 9.**
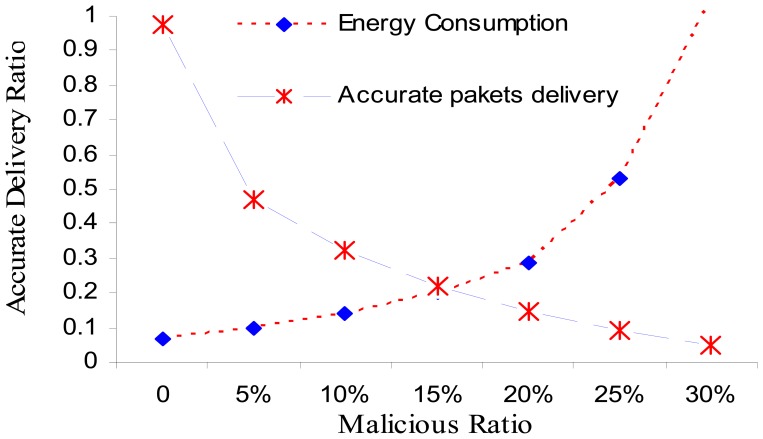
Black Hole attack run with seven scenarios implemented according to the number of malicious nodes on the network, the energy consumption of each sensor node is as follows [17]: Ea =100 pJ/bit/m2, Ee = 50 nJ/bit and Ec = 5 nJ/bit where consumed for transmitting, receiving and listening respectively. Each sensor needs to send a packet of length R = 400 bits to the cluster head on random time. Cluster head period T is set as 2,000 s and the execution time of task is set as = 0.005 s. The data packet size is 2 KB and the sensing range to 64 meters.

The simulation results of data delivery are only for the normal data delivered, provided that the network is working normally. Simulations take into consideration only special types of attacks that are not complicated to add them characteristics to our pseudo-code, like selective forwarding and black hole attacks. The simulation result compared the network with NRFP (Figure 7) and without NRFP attached (Figure 8). Figure 7 illustrates the effects observed. It allows us to measure the correct packet accurately delivered and the remaining transmission power for different scenarios of malicious nodes. The curve in Figure 8 illustrates that when the network is free from malicious nodes, 95-100% of accurate data reach safely and is real without falsification. The percentage of delivering accurate data reduces as the number of malicious nodes increases. Keeping the same conditions and simulation environments, when the malicious nodes are 30% the data delivered ratio more than 60% as shown in Figure 7 compared with less than 20% of data delivery in Figure 9 with same percentage of malicious nodes. In addition, Figure 9 shows less gradually with increasing the proportion of malicious nodes until reach to specific rate; the network stopped when the malicious nodes ratio reached more than 30% because a very low amount of accurate information reached to the sink because of the structure of the network setup.

**Figure 10.**
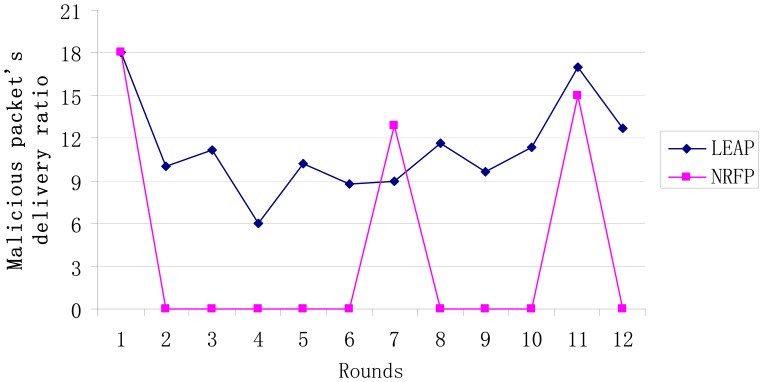
Average of malicious data rate are received.

Figure 10 shows the probability of malicious data rate are received. We assumed encryption keys have been cracked in rounds (1, 7, and 11) According to the different versions of LEAP protocol, malicious nodes are able to send data that will be acceptable by the receiver nodes and this security failure will continue for all rounds according to the characteristics of LEAP protocol. The compromised key(s) (Figure 10) affect the current round of NRFP protocol unlike the rest of the protocols, whereas the malicious node(s) will continue to work for all rounds.

On the other hand, according to the proposed algorithm the malicious nodes are able to send data that will be acceptable by the receiver nodes only in rounds (1, 7, and 11) which has been crack as we assumed. On the other rounds malicious nodes are able to send data using the old key that will be not acceptable by the receiver nodes because in every round there are new keys have been self generated by the nodes according to a key generation function imprinted in each node. The receiver nodes are ready to receive the data encrypted by new keys in new round which is the main feature of the proposed algorithm.

The advantage of NRFP models that use a single S_K_ for whole network is the decentralized communication process where establishing fast communication between nodes has the same range transmission and non-energy consumption consumed in computational process for generating a session key. It also boasts scalability to extend the network size; adding new nodes or replace the nodes that have failed without incurring significant overheads. So different applications do not require the preparation of a large number of keys as it requires changing the generated function of these keys to suit each application separately. The drawback for NRFP modules is the high risk for current round if the session key is obtained. The differences of risk between these modules are the BS-module risk for whole network in obtained round, and the risk of cluster module is only a risk for a cluster. However, the attacker will not be able to use the key for next current round. New keys are generated in the next round; therefore the attacker will not be able to use the keys.

## 9. Conclusions

In short, this paper presents a secure group communication scheme that optimizes the link layer communication of WSNs. The scheme is independent of the underlying key management architecture. Our scheme relies on clustering which divides the sensor field into control clusters with a cluster head in each cluster. We have proposed a LAFs function that efficient in establishing a secure link-layer communication. LAFs has the following properties: suitable anytime senders and receivers wish to guarantee integrity between sender and receiver, computationally very fast and very compact, accomplishes both of these properties with its reliance on a given hash function that are both fast and return compact outputs. The issue of when a new node joins and leaves the cluster was also addressed. The NRFP has two kinds of re-keying system models; the difference between these two models is that the first model uses only one session key for the whole network and uses the base station for the re-keying process periodically or when needed depend on the interrupt factor. Conversely, in the second model each cluster changes its on session key periodically or when re-keying needed, and sends the new session key to save its base station for clusters communication use.
